# Infectious Complications and Morbidities After Neonatal Bloodstream Infections: An Observational Cohort Study

**DOI:** 10.1097/MD.0000000000003078

**Published:** 2016-03-18

**Authors:** Ming-Horng Tsai, Chiang-Wen Lee, Shih-Ming Chu, I-Ta Lee, Reyin Lien, Hsuan-Rong Huang, Ming-Chou Chiang, Ren-Huei Fu, Jen-Fu Hsu, Yhu-Chering Huang

**Affiliations:** From the Division of Neonatology and Pediatric Hematology/Oncology, Department of Pediatrics, Chang Gung Memorial Hospital, Yunlin (M-HT); Department of Nursing, Division of Basic Medical Sciences and Research Center for Industry of Human Ecology, Chang Gung University of Science and Technology, Chiayi (C-WL); Department of Anatomy, College of Medicine, China Medical University, Taichung (I-TL); Division of Pediatric Infectious Disease (Y-CH); Division of Pediatric Neonatology, Department of Pediatrics, Chang Gung Memorial Hospital (S-MC, RL, H-RH, M-CC, R-HF, J-FH); and College of Medicine, Chang Gung University, Taoyuan, Taiwan (M-HT, S-MC, RL, H-RH, M-CC, R-HF, J-FH, Y-CH).

## Abstract

Few data are available on the clinical characteristics of complications and morbidities after neonatal bloodstream infections (BSIs), understood as any newly infectious focus or organ dysfunction directly related to BSIs but not occur concurrently. However, these bloodstream-associated infectious complications (BSICs) contribute significantly to increased hospital stay, cost, and final mortality.

We performed an observational cohort study of unselected neonatal intensive care unit (NICU) patients based on records in a large clinical database. All neonates hospitalized in our NICU with BSI between 2006 and 2013 were reviewed, and those who developed BSICs were analyzed to identify the clinical characteristics and outcomes. Multivariate logistic regression was used to identify independent risk factors for BSICs.

Of 975 episodes of neonatal BSI, 101 (10.4%) BSICs occurred in 93 neonates with a median interval of 3 days (range, 0–17 days) after onset of BSI and included newly infectious focuses in 40 episodes (39.6%), major organ dysfunctions after septic shock in 36 episodes (35.6%), and neurological complications after meningitis or septic shock in 34 episodes (33.7%). All patients with BSICs encountered various morbidities, which subsequently resulted in in-hospital death in 30 (32.3%) neonates, critical discharge in 4 (4.3%), and persistent sequelae in 17 (18.3%). After multivariate logistic regression analysis, independent risk factors for BSICs included initial inappropriate antibiotics (odds ratio [OR], 5.54; 95% confidence interval [CI], 3.40–9.01), BSI with septic shock (OR, 5.75; 95% CI, 3.51–9.40), and BSI concurrent with meningitis (OR, 9.20; 95% CI, 4.33–19.56).

It is worth noting that a percentage of neonates with BSI encountered subsequent sequelae or died of infections complications, which were significantly associated with initial inappropriate antibiotic therapy, septic shock, and the occurrence of meningitis. Further investigation is warranted to decrease the occurrence of BSICs due to their significant contribution toward final mortality.

## INTRODUCTION

Bloodstream infection (BSI) is a common complication of neonates hospitalized for a long period in the neonatal intensive care unit (NICU) after surviving the early problems of prematurity and is associated with high mortality and morbidity rates.^1–3^ Although most studies regarding outcomes of neonatal BSI focused on sepsis-attributable mortality, early mortality, or in-hospital mortality,^4–8^ recent studies have included the long-term outcomes in neonates with BSI or late-onset sepsis, such as worse neurodevelopmental outcomes and growth delay.^9,10^ Although most neonatal BSIs respond well to antibiotic therapy, a certain percentage of neonates suffer from bloodstream associated infectious complications (BSICs) and this issue has not yet been well studied.

BSICs include newly infectious focus, subsequent organ dysfunction, and any uneventful complications following an episode of BSI. These morbidities deserve greater attention not only because they are associated with a longer hospital stay, greater cost, and more suffering, but also because they potentially can lead to a subsequent new episode of BSI.^11,12^ Furthermore, our previous studies have concluded that infectious complication is the independent risk factor for mortality in neonates with gram-negative bacteremia.^13,14^ Although case reports and small case series have reported a variety of neonatal BSI-related complications, including orchitis, epididymitis, pericarditis, septic pulmonary embolism, or subdural empyema,^1,15–18^ no previous report has described the characteristics of BSICs in a population of critically ill neonates. We therefore conducted this study to investigate the impact of BSICs on outcomes using a large clinical database of neonates with BSI.

## PATIENTS AND METHODS

### Study Design, Setting, and Patients

We conducted a retrospective cohort study on the prospectively collected database, which was updated by the neonatologist specialist every weekday since 2003 and contained information about patients’ basic demographic characteristics, records of all complications of prematurity and all nosocomial infections, summary of hospital courses, and final diagnosis of all patients. The study was conducted at the NICU of Chang Gung Memorial Hospital (CGMH), a university-affiliated tertiary-care hospital, which has a total capacity of 49 beds equipped with mechanical ventilator and 28 beds with special care nurseries. All neonates with BSI between January 2006 and December 2013 were identified by using the clinical laboratory records and our database. This study was approved by the institutional review board of CGMH, with a waiver of informed consent because all patient records/information were anonymized and deidentified prior to analysis. The reference number of the ethical approval is CGMH 102-0013B.

### Case Finding and Study Definitions

Hospital records of all neonates with BSI were retrieved for detailed chart review by 2 investigators (Dr S-MC and Dr J-FH) for evidence of complications after BSI. In order to discriminate from concomitant infection and BSI-associated presenting symptoms and/or signs, BSICs were defined as:

- identification of any newly infectious focus directly related to BSI, and occurred at least 24 hours after the onset of BSI, which was defined at the time of the blood culture was obtained; or
- evidence of major organ damage that appeared between 12 hours and 3 days after onset of the BSI episode and lasted for >24 hours.

Other definitions applied were as the followings.

#### Bloodstream Infection (BSI)

Criteria from the Centers for Disease Control and Prevention (CDC) were applied to define neonatal BSI.^19^ We considered the following microorganisms including *Corynebacterium*, *Propionibacterium*, *Penicillium*, and diphtheroids to be contaminants and excluded them from analysis. For episodes of coagulase-negative *Staphylococci* (CoNS) BSI, the strict criteria from the CDC were applied and the episode was reviewed to avoid cases of contamination.^19^ Empirical antibiotic therapy was considered inappropriate if the antimicrobial regimens did not contain at least 1 antibiotic active in vitro against the infecting microorganisms within 24 hours of blood culture collection.

In our NICU, empirical antibiotics were initially prescribed for the coverage of both gram-positive and gram-negative pathogens, usually oxacillin or vancomycin (for late-onset sepsis) or ampicillin (for early-onset sepsis) plus gentamicin or cefotaxime, once an episode of BSI was suspected. The attending physician would modify antimicrobial regimens after the result of blood culture was informed, mostly according to the antibiotic susceptibility patterns.

#### Chronic Medical Conditions

To identify preexisting chronic medical conditions that might predispose neonates to serious infectious complications, we reviewed all hospital courses of neonates with BSI. All comorbidities of prematurity including respiratory distress syndrome (RDS), bronchopulmonary dysplasia (BPD), intraventricular hemorrhage (IVH), necrotizing enterocolitis (NEC), short bowel syndrome, and persistent pulmonary hypertension of newborn (PPHN) were documented based on the latest updated diagnostic criteria in the standard textbook of neonatology.^20^ Congenital anomalies in this study included all genetic disorders, chromosome abnormalities, documented and undocumented syndromes, and metabolic disorders, but excluded simple cleft palate or polydactyly.

BSI-related complications (BSICs) included the following:

1. Newly infectious focus included orchitis, any abscess formation, empyema, pericarditis, osteomyolitis, septic arthritis, ventilator-associated pneumonia (VAP, based on the strict diagnostic criteria of CDC^19,21^), and definite NEC (≥stage IIA, based on modified Bell criteria^22^) that were proven directly related to the episode of BSI.
2. Neurological complications following neonatal BSI with meningitis included: postinfectious encephalopathy (based on definitions from CDC^19^) – patients with changes in the level of consciousness lasting >24 hours; seizure disorder – patients without an underlying seizure disorder, perinatal insults, or brain pathology that occurred within 5 days of onset of BSI; and any neuroimaging studies-documented ventriculomegaly, hydrocephalus, encephalomalacia, or brain infarction that occurred in a neonate without previous IVH, perinatal insults, or brain pathology and was proven subsequently to meningitis.
3. Persistent organ damage included acute renal failure with/without the requirement of hemodialysis, acute respiratory distress syndrome (ARDS), disseminated intravascular coagulopathy (DIC), short bowel syndrome after surgical treatment of NEC or peritonitis, secondary pulmonary hypertension with/without cor pulmonale, and multiorgan failure.
4. Other miscellaneous items included peripheral limb gangrene, septic emboli, renal fungal ball (detected by ultrasound examination), venous thrombosis, and intracardiac vegetation, documented by abdominal sonography or duplex Doppler ultrasonography. These complications were confirmed by at least 2 independent neonatologists.

### Data Collection

In addition to the prospectively collected database, a detailed chart review was performed by the attending physicians of all the neonates with bacteremia using the electronic medical record to identify these clinical variables: presence and duration of central venous catheter (CVC), mechanical ventilators, and total parenteral nutrition; details of treatment, including antimicrobial therapies; results of all cultures from sterile sites, abscess, empyema, cerebrospinal fluid (CSF), or endotracheal aspirates; findings of all imaging studies; and clinical outcomes.

### Statistical Methods

We expressed continuous variables as medians and interquartile ranges and categorical variables were summarized with frequencies. Categorical variables were analyzed by theχ^2^ or the Fisher exact test, whereas continuous variables were analyzed by the Mann–-Whitney *U*-test. A *P* value <0.05 was considered to be statistically significant (2-tailed).

To estimate the incidence of BSIC, all neonates with BSI during the study period were retrieved for comparison, but episodes of fulminant neonatal BSI with early mortality within 48 hours of onset were excluded. We derived the unadjusted and adjusted odds ratios (ORs) and 95% confidence interval (95% CI) to examine the risk factors for the development of complications after BSI. Multivariate logistic regression analysis for independent factors potentially associated with infectious complications after BSI included all statistically significant variables with *P* < 0.1 in univariate analysis. All statistical analyses were performed using SPSS version 15.0 (SPSS, Chicago, IL).

## RESULTS

Among 5010 neonates hospitalized in our NICU over an-8 year period, 768 neonates had a total of 930 episodes of late-onset sepsis (blood culture obtained after 72 hours of life) and 64 episodes of early-onset sepsis (blood culture obtained ≤72 hours). A total of 57 episodes of meningitis were also noted during the study period. After excluding 19 episodes of BSI (12 episodes of neonatal BSI had missing data, and 7 neonates with BSI were transferred to another hospital), 101 of 975 (10.4%) episodes of BSI in 93 neonates were followed by a variety of BSICs. All BSICs, causative or related microorganisms, and onset of BSICs from BSI are summarized in Table 1.

**TABLE 1:**
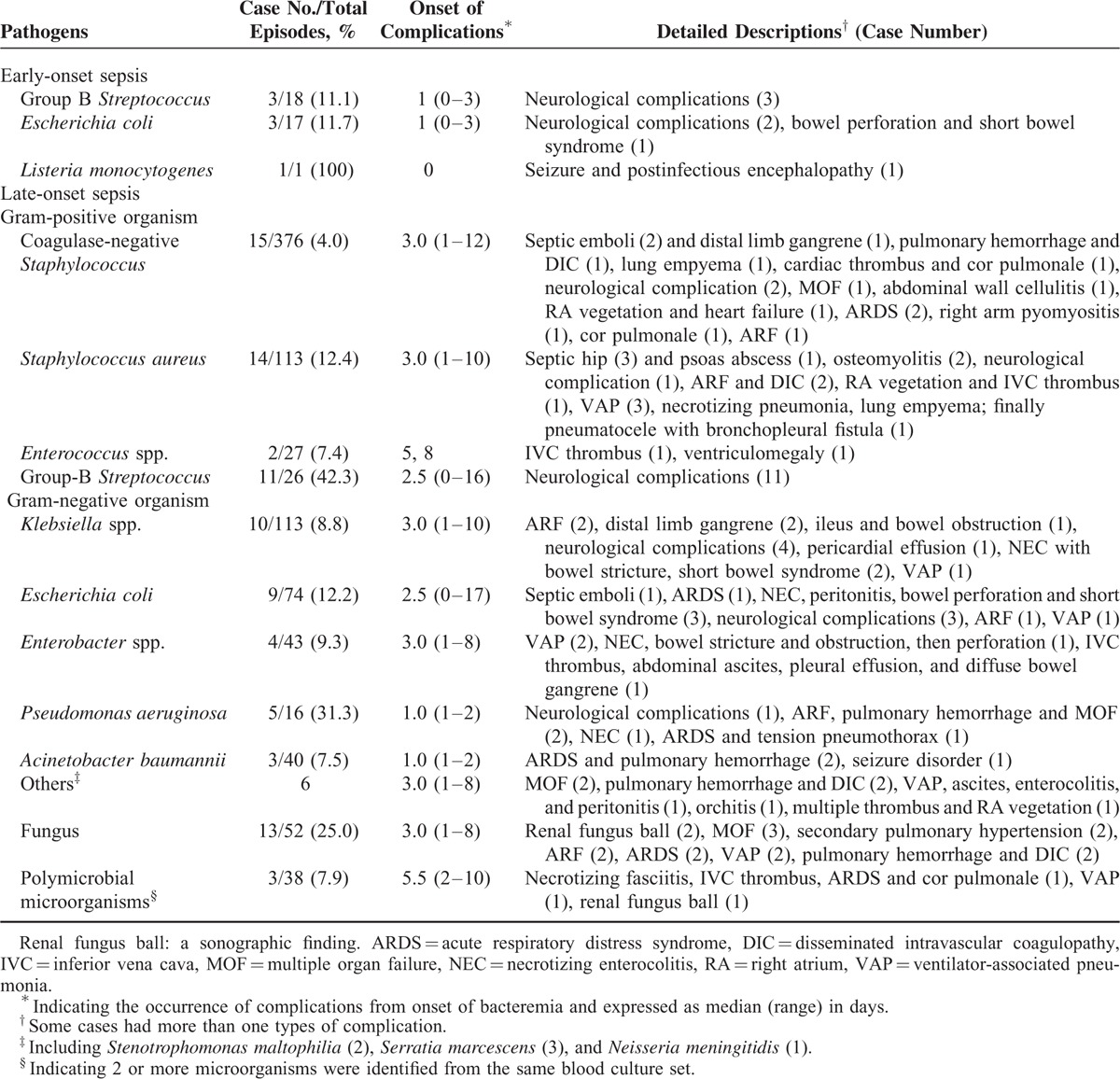
Pathogens, Frequency, and Summary of All Neonatal Bloodstream Infections Associated Complications and Morbidities in the Neonatal Intensive Care Unit

One of the major BSICs was major organ dysfunctions related to severe sepsis or septic shock. Among a total of 42 BSI episodes with septic shock in this cohort, 36 (85.7%) had major organ hypoperfusion or hypoxia-associated complications, including secondary pulmonary hypertension (n = 7), ARDS (n = 7), cor pulmonale (n = 4), acute renal failure (n = 13), severe sepsis-related neurological complications (n = 6), and pulmonary hemorrhage and DIC (n = 6). Eight patients had progressive multiple organ failure following BSI. Except for broad-spectrum antibiotics, cardiac inotropic agents, and aggressive transfusions of blood components, inhalation of nitric oxide (iNO) and hemodialysis were administrated in 10 and 1 cases, respectively. A total of 27 (75.0%) of these 42 bacteremic episodes with septic shock and BSICs finally led to fatal outcomes, including 8 due to multiple organ failure, 11 due to cardiopulmonary failure, and 8 due to additional episodes of nosocomial infections. Two cases were discharged on parent request in critically ill conditions.

Neurological complications were noted after 34 (33.7%) episodes of BSI with a median interval of 3 days (range, 0–17 days) from the onset of BSI. Seizure was the most common manifestation (22/34, 64.7%), followed by hydrocephalus (n = 15, 44.1%) and ventriculomegaly (n = 14, 41.2%). Seven patients had seizures as the initial symptom of BSI. Group B *Streptococcus* (GBS) was the most common pathogen associated with neurological complications (n = 14, 41.2%), followed by *Escherichia coli* (n = 6, 17.6%). The clinical manifestations and pathogens associated with neurological complications after BSI are shown in Table 2.

**TABLE 2:**
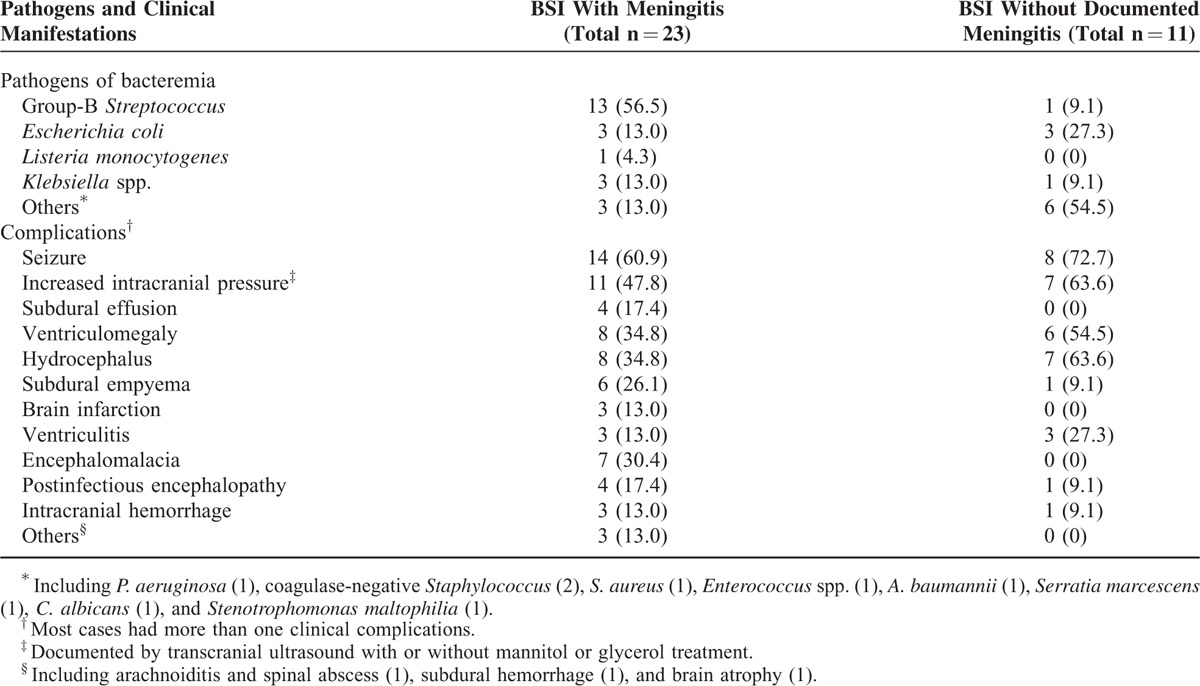
Summary of Neurological Complications After Neonates With Bloodstream Infections (BSIs)

Approximately two-thirds (23/34, 67.6%) of the patients with neurological complications had CSF culture-confirmed meningitis. Among 11 cases without positive CSF culture, 5 cases had evidences of central nervous system infection, including pleocytosis, decreased glucose and elevated protein concentrations in the CSF specimen and ventriculitis (n = 3) shown on brain ultrasonography. Their negative CSF culture may have resulted from the delayed lumbar puncture performed after the administration of systemic antibiotics. The remaining 6 cases developed postinfectious brain insult after severe sepsis or septic shock, and manifested as seizure, brain swelling, ventriculomegaly, or hydrocephalus after bacteremia. In addition to systemic antibiotics, 15 patients received surgical interventions including ventriculo-peritoneal shunting, extraventricular drainage, or subdural drainage. All neonates with seizures had evidence of focal or multifocal epileptiform discharge on the electroencephalogram examination and required anticonvulsant therapy. In 7 neonates with subdural empyema, arachnoiditis, or spinal abscess, only 4 received surgical intervention; the other 3 infants had diffuse brain injury or encephalomalacia, and were discharged in critical condition on request. Overall, in 34 neonates with neurological complications, 17 were stable (including 5 receiving oral anticonvulsant therapy), 6 died, and 11 had persistent sequelae (including 3 who were discharged in critical condition).

Forty (39.6%) of all BSICs were newly infectious focuses; the most common was VAP (n = 13, 34.2%), followed by NEC or peritonitis (n = 7), subdural empyema (n = 7), septic hip arthritis (n = 4), and osteomyelitis (n = 2) (Table 3). The median time from the onset of BSI to newly infectious focuses was 3 days (range, 1–7 days). Symptoms/signs of VAP included new patch consolidation or generalized haziness on the chest x-ray, increased secretion from endotracheal tubes, increased oxygen requirement, and abnormal laboratory findings including elevated C-reactive protein, leukocytosis or leukopenia, and positive bacterial culture from endotracheal aspirates. One case had necrotizing pneumonia with pleural effusion and lung empyema, and subsequently pneumatocele with bronchopleural fistula formation was noted. Seven patients with concomitant NEC (n = 3) or NEC and peritonitis following bacteremia (n = 4) received surgical intervention. They suffered from complications of short bowel syndrome (n = 5), bowel stricture (n = 2), and adhesion ileus (n = 1). All the cases of septic hip arthritis and osteomyelitis were associated with methicillin-resistant *S. aureus* (MRSA) BSI. One case had psoas muscle abscess 9 days after septic hip arthritis and required multiple surgical interventions.

**TABLE 3:**
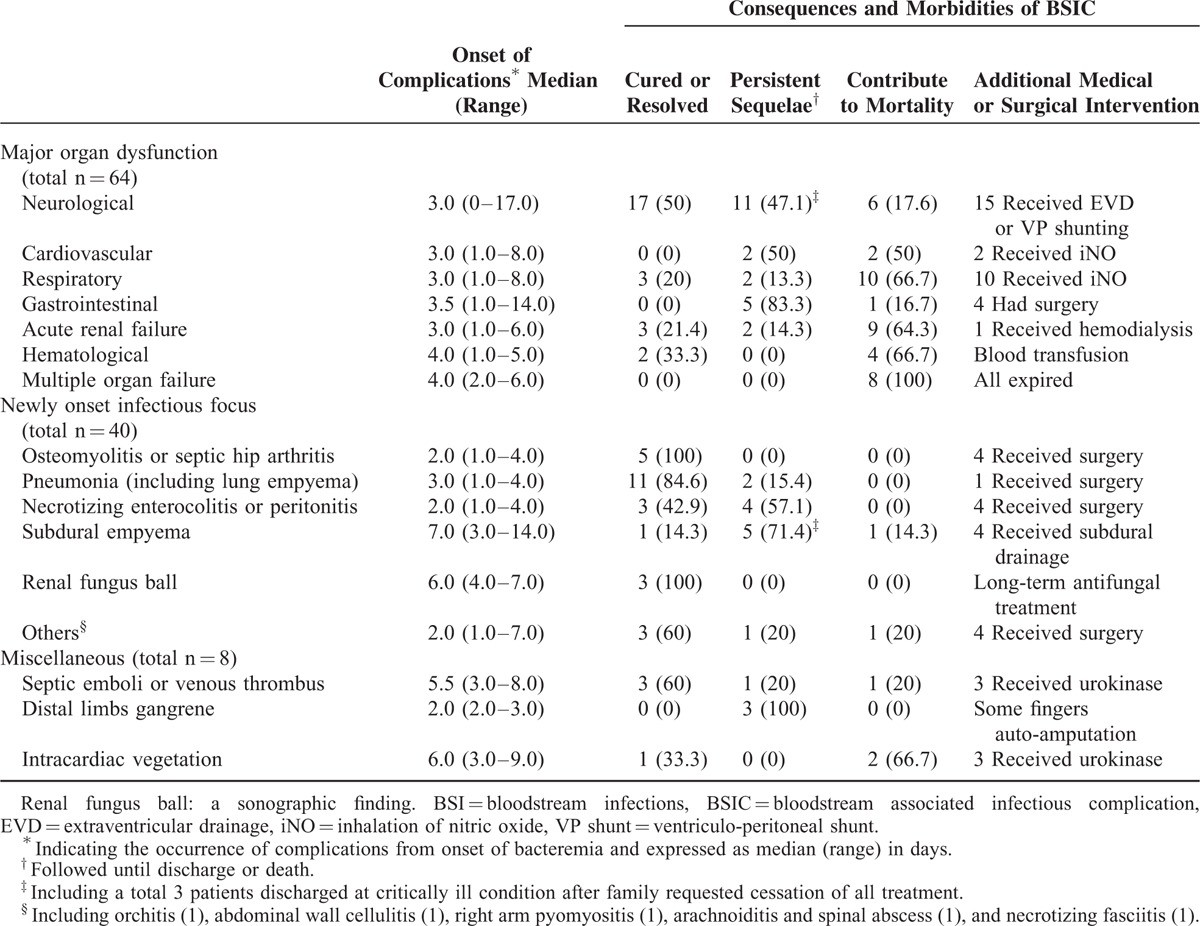
Spectrum of all BSIs Associated Complications and the Morbidities

Renal fungal ball was detected in 3 neonates with persistent fungemia by the ultrasonography examination. They had hematuria, persistent thrombocytopenia, abdominal distension, and decreased urine output. All 3 cases resolved after long-term antifungal treatment. An additional 4 patients experienced certain unusual infectious focuses after BSI, including orchitis (*N. meningitidis*), abdominal wall cellulitis (CoNS), pyomyositis (*S. aureus*), and necrotizing fasciitis (polymicrobial pathogens) in one each. All these patients had the same pathogens of initial BSI identified from the lesion sites, even 7 days or later after the onset of BSI. All of these patients recovered uneventfully.

The last category of complications consisted of septic emboli (n = 5), IVC thrombi (n = 3), and right atrium (RA) vegetation (n = 3). Urokinase and systemic antibiotics were administrated for these cases. Three of 5 patients with septic emboli encountered gangrenous changes in distal limbs with subsequent autoamputation of several fingers. IVC thrombi were incidentally identified in 2 cases, without significant manifestations. The other case with IVC thrombus developed diffuse bowel gangrene, ascites, and subsequently died. Two of 3 patients with RA vegetation experienced right heart failure and both eventually died, and the other case with RA vegetation resolved spontaneously within 6 weeks.

**FIGURE 1:**
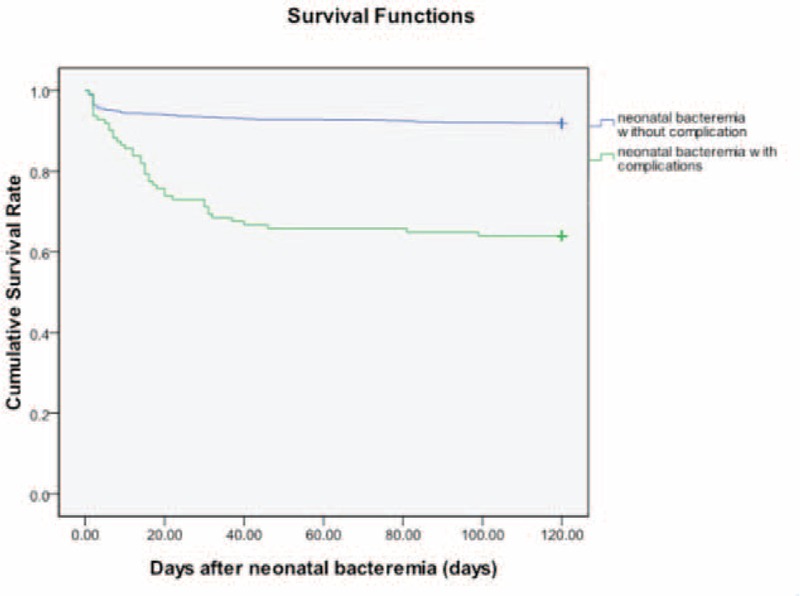
Survival following neonatal bloodstream infections with complications versus those without complications. The y-axis is the proportion surviving (*P* < 0.001 by log rank test).

Overall, among these 93 neonates with a total of 101 episodes of BSI followed by BSICs, 30 (32.3%) neonates eventually died. All the others had various degrees of morbidities and included 4 (4.3%) with critical discharge on request, 17 (18.3%) with persistent sequelae and 42 (45.2%) with eventually stable status. On the basis of a calculated 253,644 neonate-hospital days of observation, we estimated that the incidence of BSIC was 4.0 cases per 10,000 neonate-hospital days. Neonates with BSIs who experienced infectious complications had a significantly higher rate of mortality (34/93, 36.6%) than those without infectious complications (56/656, 8.5%) (*P* value < 0.001 by log rank test) (Figure 1).

Possible risk factors for infectious complications after BSI are shown in Table 4. The occurrences of BSICs were not related to gestational age or birth body weight. BSI caused by GBS or fungus, and the presence of CVC at the onset of BSI was significantly associated with a higher risk of the occurrence of BSICs. However, after adjusting for gestational age and various factors, multivariate analysis revealed that inappropriate antibiotics administered within 24 hours after onset of BSI (OR, 5.54; 95% CI, 3.40–9.01), BSI with septic shock (OR, 5.75; 95% CI, 3.51–9.40), and BSI with meningitis (OR, 9.20; 95% CI, 4.33–19.56) were independently associated with the development of complications.

**TABLE 4:**
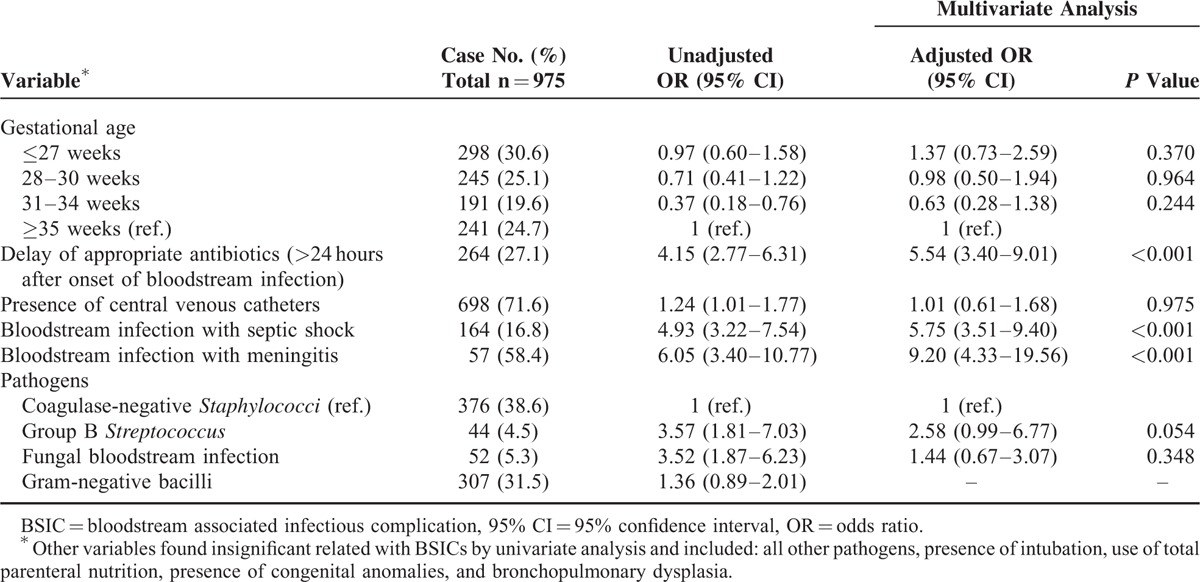
Multivariate Analysis of Independent Risk Factors for BSICs

## DISCUSSION

Results from the present study indicated that for NICU patients, the incidence of BSICs was approximately 10.4% among all neonatal BSI episodes and 4.0 cases per 10,000 neonate-hospital days. The median time from the onset of neonatal BSI to the occurrence of BSICs was 3 days (range, 0–17 days), depending on the characteristics of BSICs and the causative pathogens. Initial inappropriate antibiotic treatment, BSI with meningitis, and septic shock were independent risk factors associated with the development of BSICs.

The present study revealed that the BSICs were highly associated with the causative pathogen of the initial BSI. For example, GBS was associated with 31.8% (18/57) cases of meningitis, and the subsequent neurological complications (14/34, 41.2). Septic hip arthritis and osteomyelitis were associated with MRSA and gram-negative bacilli may potentially cause NEC or other GI tract complications. For intracardiac vegetation or IVC thrombus, the common pathogens were CoNS or MRSA. The onset of BSICs after septic shock or meningitis always occurred within 1 week after the onset of BSI, but certain rare complications, such as orchitis, arachnoiditis, spinal abscess, and femoral neck fracture after septic hip arthritis, may occur after a relatively longer interval from the onset of BSI.

Most of the cases with neurological complications resulted from meningitis. We observed that 40.4% of the neonates with BSI complicated with meningitis had neurological complications. Approximately one-third of neonatal GBS meningitis or 7.8% to –13.8% of neonatal GBS sepsis cases were previously reported to have neurological sequelae at discharge.^23–25^ This study noted a similar percentage of neurological sequelae in neonates with GBS sepsis or meningitis, but a much higher rate of neurological complications following GBS meningitis (14/18, 77.8%) and GBS sepsis (14/44, 31.8%). Besides, we also found late-onset GBS sepsis was significantly more likely to be associated with neurological complications than early-onset GBS sepsis (42.3% vs 11.1%, *P* < 0.001).

The majority of newly infectious focuses were supposed to be the focal suppurative complications of initial BSI, and some of them have been published as case reports or case series in the literature.^8–10^ Persistent BSI may account for some infectious complications. Chapman et al have found that the duration of persistent BSI is highly correlated with the focal complication.^26–29^ In this study, we found inappropriate antibiotics within 24 hours after onset of BSI was the independent risk factor of BSICs, which can be explained by the fact that it may cause persistent BSI and subsequent focal complications. However, unnecessary broad-spectrum antibiotic use is associated with emergence of antibiotic resistant pathogens in the NICU.^13,30^ Therefore, further studies regarding early identification or accurate prediction of antibiotic-resistant microorganisms may allow optimization of appropriate antibiotic administration and reduction of BSICs.

Risk factors associated with the development of septic thromboembolism included critically ill patients, presence of CVC, and MRSA BSI.^10,31,32^ Based on our experience, early diagnosis and prompt thrombolysis of significant lesions contributed greatly to successful treatment. Early diagnosis of venous thrombus or vegetation relied on high suspicion, and the incidence of septic emboli or thrombus was expected to be higher than what we observed, since abdominal or duplex Doppler ultrasonography was not routinely performed in each patient with BSI. However, only a few cases of successful thrombolysis of neonatal thrombosis have been reported in the literature.^33,34^

Although this study provides useful information about complications after BSI, it has several limitations. First, in cases with underlying neurological comorbidities of prematurity or congenital central nervous system malformation, it is difficult to attribute neurological complications to underlying conditions or BSI with meningitis. Second, the definition of VAP after BSI is somewhat arbitrary, and sometimes we failed to prove their association or exclude the possibility of superimposed infection. Finally, the study was conducted in only 1 medical center in Taiwan, and therefore the incidence and spectrum of disease could be different in other areas and could be affected by different treatment strategies.

## CONCLUSIONS

Based on our cohort study, complications after BSI were not rare in the NICU and potentially caused mortality and morbidities. BSI episodes with septic shock or combined meningitis were independent risk factors for the occurrence of BSICs. Because of the significant contribution of BSICs toward an adverse final outcome, further studies regarding infection control strategies in order to reduce the BSI incidence and morbidities after BSI are worth consideration in the NICU.
