# Infectious Complications and Morbidities After Neonatal Bloodstream Infections: An Observational Cohort Study: Erratum

**DOI:** 10.1097/01.md.0000483237.10561.2b

**Published:** 2016-05-13

In the article “Infectious Complications and Morbidities After Neonatal Bloodstream Infections: An Observational Cohort Study”,^1^ which appeared in Volume 95, Issue 11 of *Medicine*, the ethical approval reference number in the “Study Design, Setting, and Patients” section was misreported. The article has since been corrected online.
